# Cloning, Expression, Purification and Crystallization of the PR Domain of Human Retinoblastoma Protein-Binding Zinc Finger Protein 1 (RIZ1)

**DOI:** 10.3390/ijms9060943

**Published:** 2008-06-02

**Authors:** Wanpeng Sun, C. Ronald Geyer, Jian Yang

**Affiliations:** 1 College of Pharmacy and Nutrition, University of Saskatchewan, 110 Science Place, Saskatoon, Saskatchewan, S7N 5C9, Canada E-mail: w.sun@usask.ca. E-mail: jian.yang@usask.ca; 2 Department of Biochemistry, College of Medicine, University of Saskatchewan, 107 Wiggins Road, Saskatoon, Saskatchewan, S7N 5E5, Canada E-mail: ron.geyer@usask.ca

**Keywords:** tumor suppressor, Yin-Yang regulation, zinc finger, affinity chromatography, protein crystallization

## Abstract

Through alternative promoter usage, human retinoblastoma protein-interacting zinc finger gene *RIZ* encodes two different protein products, RIZ1 and RIZ2, which have been identified to be a tumor suppressor and a proto-oncoprotein, respectively. Structurally, the two protein products share the same amino acid sequences except that RIZ2 lacks an N-terminal PR domain with methyltransferase activity. Previous studies have shown that over-expression of RIZ2 is usually associated with depressed RIZ1 expression in different human cancers. It is generally believed that RIZ1 and RIZ2 regulate normal cell division and function using a “Yin-Yang” fashion and the PR domain is responsible for the tumor suppressing activity of RIZ1. In order to better understand the biological functions of the PR domain by determining its three-dimensional crystal structure, we expressed, purified and crystallized a construct of the PR domain (amino acid residues 13–190) in this study. The maximum size of the needle-shaped crystals was approximately 0.20 × 0.01 × 0.01 mm.

## 1. Introduction

Human retinoblastoma protein-interacting zinc finger gene, *RIZ*, was first identified from a functional screening for retinoblastoma tumor suppressor binding genes [1, 2]. It is located on the distal short arm of chromosome 1 (1q36), a region which harbors several other tumor suppressors and is frequently deleted in human cancers [3–9]. Using two different promoters, the *RIZ* gene encodes two different protein products, RIZ1 and RIZ2, which regulate normal cell division and function in a Yin-Yang fashion [10–14]. RIZ2, a proto-oncoprotein, promotes cell division; whereas RIZ1, a tumor suppressor, arrests cells in the G2/M phase of the cell cycle and induces apoptosis [12–14]. Silencing of RIZ1 expression is associated with increased RIZ2 expression. This inverse correlation in RIZ expression has been observed in human hepatoma, leukemia, malignant lymphoma, breast cancer and colorectal cancer [15–19]. Delivery of RIZ1 *via* viral vector suppresses the growth of hepatoma and other cancer cells [12, 17].

RIZ1 and RIZ2 have identical amino acid sequences except that RIZ2 lacks the N-terminal PR domain region (200 amino acids, a subclass of zinc finger proteins present in PRDI-BF1/Blimp-1 and RIZ1) [10, 12, 13]. Therefore, the PR domain is likely responsible for the tumor suppressing activity of RIZ1. *In vitro* studies showed that the PR domain interacts with a PR binding motif (PRB) located in the C-terminal region of both RIZ1 and RIZ2 [11, 20, 21]. This interaction might play an important role in the “Yin-Yang” regulation between RIZ1 and RIZ2. Structurally related to the SET domain of the chromatin-associated proteins involved in gene expression, the PR domain is a C2-H2 type of zinc finger protein possessing histone H3K9 methyltransferase activity [14, 22]. Currently, more than 20 PR domains and 30 SET domains are identified in human [22, 23]. Amino acid sequence identity is about 45% among the PR domains, 50% among the SET domains, and 20% between the PR and SET domains [23]. In 2005, Derunes *et al*. first reported the crystallization of a construct (amino acid residues 1–161) of the PR domain [22]. However, the crystallization conditions were not released and the crystal structure of the construct has not yet been reported. This research group however reported NMR structures of the same construct (re-classified as a SET domain) earlier this year [24]. Despite the availability of the NMR structures, our current crystallographic investigation on the PR domain was continued because our PR domain construct (amino acid residues 13–190) is much longer in the C-terminus and valuable information may be deduced for residues 161 to 190 from the crystal structure. In addition, comparison between the crystal structure and the NMR structures of the PR domain might reveal some key features that are crucial for its *in vivo* biological functions.

## 2. Results and Discussion

### 2.1. Cloning and expression of DNA fragment encoding the PR domain

**Figure 1.**
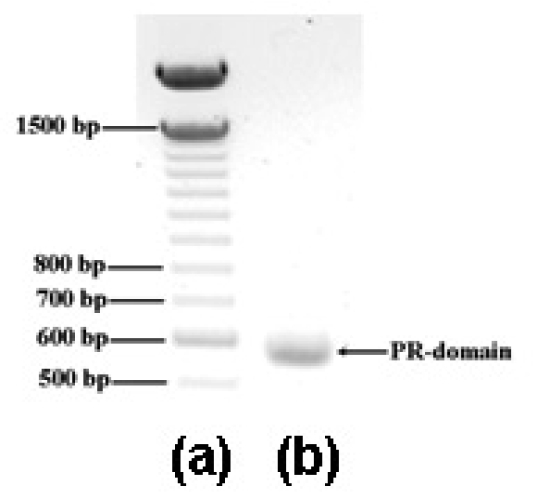
Cloning of the gene fragment encoding the PR domain of RIZ1. Lanes (a) and (b) were the nucleotide markers and the PCR product, respectively.

As described below in the Experimental Section, a DNA fragment encoding the PR domain of RIZ1 (amino acid residues 13–190) was cloned by the polymerase chain reaction (PCR) using a plasmid DNA containing the full-length *RIZ* gene as the template in order to characterize the PR domain by the X-ray protein crystallography. The PCR product was confirmed to have 534 bp (Figure 1) and share 100% sequence identity with gene fragment encoding the designed PR domain construct by DNA sequencing at the Plant Biotechnology Institute, National Research Council (Saskatoon, Saskatchewan, Canada). Ligation of the PCR product to the expression vector pET-30a(+) at the *Nde*I and *Xho*I sites produced plasmid pET30/PR. Transformation of the plasmid pET30/PR into *Escherichia coli* (*E. coli*) BL21(DE3) cells expressed C-terminal His_6_-tagged recombinant PR domain. Over-expression of the PR domain was induced by isopropyl β-D-1-thiogalactopyranoside (IPTG, final concentration of 0.5 mM).

### 2.2. Purification and activity assay of PR domain

**Figure 2.**
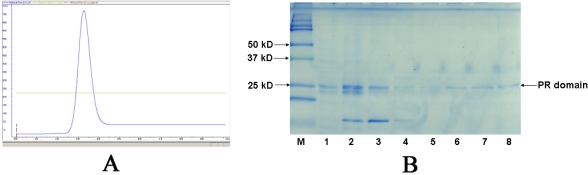
Purification of the PR domain of RIZ1. (A). Histogram showing the elution of the PR domain **on** an ÄKTA^®^ Purifier™ FPLC system. (B). SDS-PAGE (16%) of the purified His_6_-tagged PR domain. Lane M was the protein standard; whereas lanes 1 to 8 were the different collected fractions of the PR domain.

**Figure 3.**
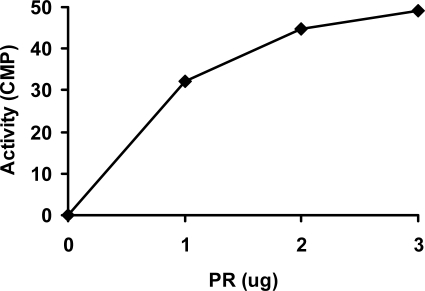
Radioactive **assay** on the methyltransferase activity of the PR domain.

In spite of the relatively low yield (1–2 mg purified protein per liter of cell culture), the recombinant PR domain was purified to homogeneous by affinity chromatography using a pre-packed 5-mL HisTrap HP column on an ÄKTA^®^ Purifier™ FPLC system (Figure 2). In contrast to normal His-tagged protein purifications, purification of the PR domain was undertaken at pH 9.3 instead of pH 7.5 to 8.0, because the PR domain did not bind to the Ni-based HisTrap column at pH 7.5 to 8.0, which was likely due to the high isoelectric point of the PR domain (calculated theoretical pI is 9.2). The purity of the PR domain in the collected fractions was examined on a 16% SDS-PAGE gel (Figure 2). The fractions (lanes 5–8) containing pure PR domain were combined, concentrated to 1 mg/mL, and stored at −80ºC. The apparent molecular weight for the PR domain was 24 kDa as determined from the SDS-PAGE gel, consistent with the calculated molecular weight. In order to determine whether the PR domain was active, we measured the methyltransferase activity of the PR domain using a P81 phosphocellulose filter paper based radioactive assay. As shown in Figure 3, the PR domain exhibited a concentration-dependent methyltransferase activity although the correlation between PR domain concentration and the methyltransferase was not quite linear. This indicated that the PR domain was in its active form suitable for X-ray protein crystallographic studies.

### 2.3. Crystallization of PR domain

**Figure 4.**
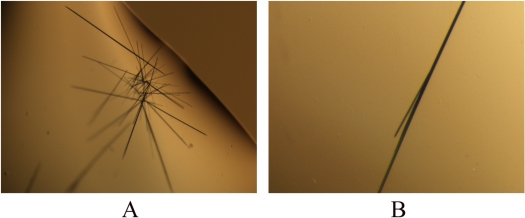
Crystals of the PR domain of human tumor suppressor RIZ1. (A). A cluster of the needle-shaped crystals. (B). A cluster from which a crystal (later identified to be a twin crystal) with the size of approximately 0.20 × 0.01 × 0.01 mm was obtained.

The initial crystallization conditions for the PR domain of RIZ1 were obtained by the hanging-drop vapor diffusion method using the commercially available Classics, Classics Lite, PEGs, PEGs II and ComPAS crystallization screening suites from Qiagen-Nextal (Mississauga, Ontario, Canada). Needle-shaped crystals appeared within 5 days from the crystallization condition with the reservoir solution containing 0.1M HEPES, pH7.5, and 4 M NaCl (Figure 4). The maximum size of the needle-shaped crystals was 0.20 × 0.01 × 0.01 mm. The crystals were capable of being stained into dark blue color using the IZIT Crystal Dye™ from the Hampton Research (California, USA), indicating that they were indeed the crystals of the PR domain. Preliminary X-ray diffraction studies at the Canadian Light Source (Saskatoon, Saskatchewan, Canada) showed that the largest crystal was a twin crystal (data not shown). Further optimization of the crystallization conditions to grow larger and thicker crystals suitable for diffraction data collection is still in progress.

## 3. Experimental Section

### 3.1. Cloning and expression of the gene fragment encoding the PR domain

Plasmid containing the full-length *RIZ* gene was kindly provided by Dr. Shi Huang of the Burnham Institute of Medical Research. Using the plasmid DNA as the template and a pair of DNA primers (forward: 5’-CATATGCGCAGACGGACAGCGGATGC-3’ with an *Nde*I restriction site; backward: 5’-CTCGAGGTAGCTGAAGTCCTTCAGCTC-3’ with an *Xho*I restriction site) with their sequences corresponding to the 5’ and 3’ ends of the gene fragment encoding the PR domain of RIZ1 (residues 13–190), respectively, we amplified a DNA fragment of the expected size by PCR reaction on an Eppendorf^®^ Mastercycler™ personal thermocycler (Eppendorf Canada, Ontario, Canada). The PCR product was cleaned using a QIAquick PCR purification kit (Qiagen Canada, Ontario, Canada) and then reacted with dATP to add adenines at both ends using the *Taq* DNA polymerase (Invitrogen Canada, Ontario, Canada). The resultant was subject to TA cloning using a TA cloning kit (Invitrogen Canada). The new PCR product was directly ligated to the TA vector pCR2.1 and transformed into *E. coli* TOP10 cells. Plasmid DNA extracted from positive transformants was digested with restriction endonucleases *Nde*I and *Xho*I. The digested DNA fragment was first confirmed to be the gene fragment encoding the PR domain by DNA sequencing and then sub-cloned into the expression vector pET30a(+) at the *Nde*I and *Xho*I sites. The resulted plasmid pET30/PR was transformed into *E. coli* DH5α cells. Plasmid DNA was extracted from positive clones on Luria-Bertani (LB)-agar plates containing kanamycin (30mg/ml) and transformed into *E. coli* BL21(DE3) cells for over-expression of the PR domain. One liter of LB plus kanamycin medium was inoculated with 1.0 mL of overnight culture from a single colony of transformed *E. coli* BL21(DE3) cells and incubated at 37 °C with shaking at 250 rpm until OD_600_ of the culture reached 0.6. Protein expression was induced by adding IPTG (final concentration of 0.5 mM) to the cell culture. Cell growth was allowed to proceed for an additional 4 hours. The cells were harvested by centrifugation at 5,000 g for 20 minutes and the pellets were stored at −80 °C.

### 3.2. Purification of the PR domain

A frozen pellet from 1 L cell culture was suspended in 40 mL of the lysis buffer (20 mM Tris-HCl, pH 9.3, 0.3 M NaCl, 10 mM imidazole, 0.5 mM phenylmethylsulphonyl fluoride (PMSF), 5 mM β-mercaptoethanol (β-ME), 1 μM pepstatin A, and 1 g/L lysozyme) and incubated with gentle rotation for 30 min. The cells were further disrupted by sonication using a Sonifier™ 150 sonicator from Branson Ultrasonics (Danbury, Connecticut, USA). The cell lysate was centrifuged at 20,000 g for 30 min. The supernatant was loaded onto a pre-packed 5-mL HisTrap HP column from GE Healthcare Canada (Baie d’Urfé, Québec, Canada). The column was washed thoroughly with about 100 mL of the wash buffer (20 mM Tris-HCl, pH 9.3, 0.3 M NaCl, 50 mM imidazole, 0.5 mM PMSF, 5 mM β-ME, and 1 μM pepstatin A) at a flow rate of 2 mL/min until the elute UV absorbance was approximately zero. The PR domain was then eluted with 20 mL of the elution buffer (20 mM Tris-HCl, pH 9.3, 0.3 M NaCl, 100 mM imidazole, 0.5 mM PMSF, 5 mM β-ME, and 1 μM pepstatin A). Purity of the PR domain in the elution fractions was examined on a 16% SDS-PAGE gel. Fractions containing pure PR domain were combined, and concentrated to 1 mg/mL, and stored at −80°C.

### 3.3. Activity assay

The activity of the PR domain was assayed by the methylation reaction. Briefly, the PR domain was added to a 20 μL reaction solution containing 16 μM radio-labeled SAM, 50 mM Tris-HCl, pH 9.0, 5 mM MgCl, 4 mM dithiothreitol (DTT), and 0.125 g/L histone H3 at different concentrations. The reaction solution was incubated at 30 °C for 1 hr before spotting onto a P81 phosphocellulose filter paper. The P81 filter paper was first washed with 10% trichloroacetic acid (15 min) three times and 95% ethanol (1 min) once, then dried, and finally added into a scintillation vial containing 5 mL scintillation cocktail. The radioactivity was measured using a scintillation counter. The result was calibrated with solvent control.

### 3.4. Crystallization of the PR domain

Crystallization of the PR domain was performed by the hanging-drop vapor-diffusion method at room temperature. Initial crystallization conditions were obtained by the sparse-matrix protocol [30]. Needle-shaped crystals were obtained within 5 days from drops containing equal-volume mixtures (2 μL:2 μL) of protein solution (1 mg/mL in 10 mM Tris-HCl, pH 7.7) and reservoir solution (4 M NaCl, 0.1 HEPES, pH 7.5) equilibrated against the reservoir solution (0.6 mL). The crystals were confirmed to be the PR domain by staining with the IZIT Crystal Dye™ from Hampton Research (Aliso Viejo, California, USA).

## 4. Conclusion

In the current study, the PR domain (amino acid residues 13–190) of human tumor suppressor RIZ1 was cloned and over-expressed. The recombinant PR domain was confirmed to be active by the methylation reaction and crystallized by the sparse-matrix protocol. The maximum size of the needle-shaped crystals was 0.20 × 0.01 × 0.1 mm. Optimization of the initial crystallization conditions to obtain large crystals suitable for X-ray diffraction is in progress.
